# Honey Bees Can Use Sequence Learning to Predict Rewards from a Prior Unrewarded Visual Stimulus

**DOI:** 10.3390/insects16040358

**Published:** 2025-03-31

**Authors:** Bahram Kheradmand, Ian Richardson-Ramos, Sarah Chan, Claudia Nelson, James C. Nieh

**Affiliations:** Section of Ecology, Behavior, and Evolution, Division of Biological Sciences, University of California San Diego, 9500 Gilman Dr, MC0116, La Jolla, CA 92093, USA; irichardson1098@gmail.com (I.R.-R.); kittenwarz@yahoo.com (S.C.); claudianelsonclaudianelson@gmail.com (C.N.); jnieh@ucsd.edu (J.C.N.)

**Keywords:** *Apis mellifera*, navigation, animal cognition, visual learning

## Abstract

Learning to anticipate upcoming events can increase fitness by allowing animals to choose the best course of action, and many species can learn sequences of events and anticipate rewards. To date, most studies have focused on sequences over short time scales such as a few seconds. Whereas events separated by a few seconds are easily learned, events separated by longer delays are typically more difficult to learn. Here, we show that honey bees (*Apis mellifera*) can learn a sequence of two visually distinct food sources alternating in profitability every few minutes. Bees were challenged to learn that the rewarded pattern was the one that was non-rewarded on the prior visit. We show that bees can predict and choose the feeder that will be rewarding upon their next approach more frequently than predicted by chance, and they improve with experience, with 64% correct choices made in the second half of their visit sequence (*N* = 320 visits by 20 different bees). These results increase our understanding of honey bee visual sequential learning and further demonstrate the flexibility of foragers’ learning strategies.

## Simple Summary

Honey bees navigate complex environments and make decisions about where to find food. This study investigates whether they can learn a sequence of events to predict future rewards. We trained honey bees to recognize a pattern in which a previously unrewarded visual cue became rewarding on the next visit. Our results showed that bees could anticipate which food source would be rewarding more often than expected by chance, demonstrating an ability to learn sequences over several minutes. Understanding how bees process information and make foraging decisions provides insights into their cognitive abilities, which are important for pollination and ecosystem health. This research highlights the cognitive flexibility of learning and decision-making in pollinators.

## 1. Introduction

Animals often perform behaviors made up of many components. They usually receive feedback after acting and use that information to choose their next action. Certain species excel at learning sequences and can solve multi-step problems, such as humans [1], other primates [2,3,4], and crows [5]. Even learning simpler sequences can be beneficial. For example, with experience, bumblebees can alter the frequency and amplitude of their buzzes to obtain floral pollen and improve foraging [6].

Sequence learning can also be thought of as reducing uncertainty by establishing the correct time-dependent connections between parts of a stimulus or within a sequence of responses [7]. It involves detecting regularities such as co-occurrence patterns or frequent transitions in sensory input [8]. However, sequences are not always composed of events that immediately follow each other. An animal’s ecology shapes the intervals between events, and these intervals can vary with season and location [9]. The delay interval is critical for sequence timing because, for longer delays, short-term memories may not sustain the necessary stimulus-evoked activity to link the unconditioned (unlearned) and the conditioned (learned) stimuli [10].

In different studies, the conditioned and unconditioned stimuli may overlap or occur back-to-back without any delay or occur after only a brief delay of a few seconds [11,12,13]. Although these short-delay protocols underlie much of our understanding of bee learning—especially associative learning that involves overlapping stimuli [14]—bees are also capable of olfactory trace conditioning, where the conditioned stimulus appears first and is followed by a delay of a few seconds before the unconditioned stimulus [15]. In most such experiments, delays are relatively short, yet in natural environments, bees commonly face more extended intervals. Bumblebees can evaluate both the amount of reward a flower produces and the timing of that production [16], suggesting that bees may have evolved the ability to remember sequences lasting several minutes [17]. Further exploring how bees handle such longer delays between the learned stimulus and the reward would provide valuable insights into their broader cognitive capacities.

We focused on visual learning because it plays a pivotal role in honey bee foraging [18]. Honey bees can successfully learn a delayed match-to-sample task by retaining a visual stimulus and then comparing it with a subsequent one, provided that the delay between the two stimuli is very short, usually less than eight seconds [19,20]. They can also learn rules that involve multiple visual stimuli presented in quick succession [21]. In addition, they can remember long-term patterns on the order of hours, such as a particular food source offering a reward in the morning and another (or the same) being rewarding in the afternoon [22]. However, such abilities might arise through circadian rhythms, or from using the sun’s position and landmarks as contextual cues [23], together with the diminishing drive to visit an unrewarding food source. Between these extremes of very short- and long-term delays, there is some evidence for visual learning with delays of minutes: navigating honey bees can form memories based upon delayed reward or punishment [24] and can learn complex movement sequences [25]. Bumblebees likewise learn routes in which they visit several flowers in a particular order, a behavior called traplining [26].

We, therefore, sought to investigate visual sequence learning in honey bees and to test their ability with a delay of several minutes between the reward and the learned visual stimulus, a condition rarely studied. We trained each bee to feed at two feeders marked with distinct visual patterns, only one of which offered a reward at a time. It is quite simple for bees to learn that one visual pattern is rewarding and that a different pattern is not [27]. The challenge lay in the fact that neither pattern remained consistently rewarding, *forcing the bees to infer that the previously non-rewarding pattern would become rewarding on the subsequent trip*. This inference required the bees to recall which pattern did not yield a reward, override the memory that this pattern was unrewarding, and then select that pattern as the rewarding one on their next trip. This is a difficult learning task and, because the bees were freely foraging and bringing food back to their hives, occurred with a delay of about five minutes between feeder visits. If they could master this task, we expected them to choose the correct feeder on each visit and make faster decisions with growing experience.

## 2. Materials and Methods

During the spring and summer of 2018 and the summer of 2019, we conducted three experiments. In total, we trained 43 European honey bees (*Apis mellifera ligustica*) from ten different colonies at the UCSD Biological Field Station in La Jolla, CA, USA. We ran one trial each day, usually between 9:00 A.M. and 3:00 P.M. Each trial had two phases: a *familiarization* phase and a *training* phase.

In the familiarization phase, we placed a single feeder (see below) in front of a colony. Once several bees began to feed on it, we moved the feeder to a white circular platform (25 cm in diameter) on a 1 m high tripod set at the same height as the colony entrance. We then slowly moved this tripod away from the colony entrance so that a few bees would continue to visit it (method from [28]). When the tripod was about 25 m from the colony, we stopped moving it and marked all arriving bees. Over the next 30 min, we recorded how often each bee returned to the feeder to identify a reliable forager, defined as the bee that visited most frequently. Because the presence of other bees can influence feeder choice [29], once we had selected and uniquely marked the focal bee with acrylic paints on its thorax, we used a manual aspirator to remove all other bees, including newly arriving recruits.

Once the focal bee had returned to the feeder at least three times without other bees present, we began the training phase. We removed the familiarization feeder, cleaned the feeder platform, and placed two clean training feeders with different visual patterns on the platform, only one of which provided a reward (see below). These two feeders were separated by 6 cm. Each feeder consisted of a feeder block covered by an overlay showing a distinct visual pattern. At the end of each trial, we captured and froze the focal bee to ensure that it was not used in subsequent trials.

### 2.1. Feeder Blocks

The feeder block was a transparent square acrylic block (3.9 × 3.9 × 1.8 cm) with a cylindrical center well (1 cm wide, 0.8 cm deep) that contained either the rewarding solution (about 1 mL of 2.5 M analytical-grade sucrose in deionized water, refilled frequently) or a non-rewarding solution (water-only or saline). We placed our feeders horizontally on the platform. This setup allowed us to switch the visual patterns more rapidly, and the bees showed no difficulty distinguishing them, as demonstrated in our positive control experiment (see below). Presenting the feeders in a horizontal orientation also mimics some natural flowers that bees visit.

We tested two different non-rewarding solutions: neutral (distilled water) or aversive (3 M NaCl in distilled water; [30]). Each bee encountered only one of these non-rewarding solutions. We adopted this strategy to enhance the motivation to avoid incorrect choices, following prior research [27,31].

### 2.2. Visual Patterns

**Figure 1:**
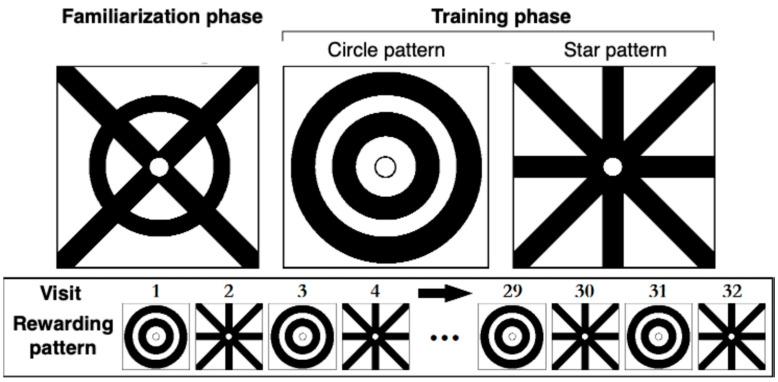
Feeders and patterns used in familiarization and training phases. The top panel shows the visual patterns drawn on clear overlays and placed over the feeder blocks. The pattern on the left (circle-cross) was used during the *familiarization* phase, and the two patterns on the right were used during the *training* phase. The bottom panel illustrates the sequence of the rewarded pattern on each visit.

We used different patterns for the familiarization phase and the training phase. We drew these patterns using a black marker (Sharpie Magnum, Model #: H-384BL, Sharpie, Atlanta, GA, USA) on square plastic overlays of transparent polyester film (Staples transparency film, Model #: 21828, Staples, Framingham, MA, USA), each measuring 3.9 cm × 3.9 cm. Each overlay also had a small (4 mm diameter) central hole that bees could not enter but could reach through with their proboscis to drink. Because *A. mellifera* exhibits preferences for certain colors in choice experiments [32], we used only black-and-white patterns. For the familiarization phase, we used a circle-cross pattern (Figure 1). For the training phase, each bee was presented with two patterns: two concentric circles and a star. Similar patterns have been commonly used in honey bee research [19,28] because bees can easily recognize concentric and radial patterns [33,34].

### 2.3. The Sequence Experiment

Bees needed to learn that the visual pattern *not associated* with reward in the previous visit would become rewarding in the subsequent visit. They could not simply associate one pattern with a reward. In the training phase, bees (*N* = 20) therefore experienced a sequence of alternating patterns paired with a reward (Figure 1, bottom panel). Once a bee was satiated and flew back to the colony, we removed and cleaned the overlays, then placed them on the opposite feeder block, thus switching which block bore the rewarding pattern. We define a trial as a training session in which a bee repeatedly visits the feeder array. Each bee was thus used in only one trial. Bees usually visited from 10:00 AM to 2:00 PM, and thus there were usually 32 visits per bee per trial. We conducted only one trial per day.

Honey bees are sensitive to scents and frequently scent-mark profitable food sources. To prevent any biases from scent marking, such as cuticular hydrocarbon footprints [35], we covered the platform with a clean white paper towel and replaced it every eight visits (based on preliminary observations). We also cleaned the overlays after each visit by dipping them in fresh water and wiping them with a clean paper napkin. We replaced the feeder blocks every eight visits, although bees rarely made contact with them or the platform directly. We did not use ethanol to remove scents; however, to test for any biases arising from residual odors, we ran a negative control experiment (*see below*), which revealed no evidence of scent-based discrimination.

To eliminate additional visual cues, both feeders always contained the same volume of liquid. Because bees can use landmarks to find food and typically arrive from the same direction, we randomly rotated the feeders among four positions (north, south, east, or west) so that the rewarding feeder, on average, occupied each position equally across visits. We chose randomly among the four cardinal compass directions so that no higher sequence could interfere with our simple alternating sequence. Thus, the only consistent information that bees could use to choose the rewarding feeder was the pattern on the overlay and its position in the alternating sequence. If bees learned to use these visual patterns and to recognize the alternating reward sequence, we predicted that they would outperform chance in selecting the rewarding feeder. To rule out alternative explanations, we conducted two control experiments.

### 2.4. Negative Control Experiment for Scents

To test if bees were relying on odor cues from the sugar solution or their scent marks, we conducted a series of negative control trials (*N* = 11). We used two feeders, but both feeder blocks bore identical visual patterns, the circle-cross design from the familiarization phase (Figure 1). In this experiment, the reward was 2.5 M sucrose solution, and the non-reward was water. We followed the same procedures as in the main sequence experiment to remove olfactory, visual, and social cues. If bees could smell the sucrose solution or their scent marks after the odor removal procedure, they should choose the rewarding feeder > 50% of visits.

### 2.5. Positive Control Experiment for Novelty-Seeking

Honey bees are *flower constant*, meaning they typically continue visiting the same flower species for an extended period as long as it remains profitable [36]. However, when a familiar food source becomes unpredictable, some bees seek out alternative sources. Such behaviors can be described as *win-stay*, where foragers return to a recently profitable location, or win-shift, where foragers abandon a location that they have just depleted [37]. If the pseudorandom rotation of feeder locations between visits led bees to perceive these feeders as unreliable, the observed pattern of alternating choices could simply be a result of novelty seeking or win-shifting.

To test for such potential novelty seeking, we performed a series of positive control trials (*N* = 12). Each trial presented bees with two feeders bearing the same distinct patterns used in the sequence experiment (Figure 1). One feeder always contained the 2.5 M sucrose solution, while the other always contained distilled water. For half of the bees, the star pattern was rewarding; for the other half, the circular pattern was rewarding. We used the same procedures as in the sequence experiment to eliminate olfactory, visual, and social cues. Instead of recording 32 visits per bee, we recorded 20 visits, because prior research has shown that bees can rapidly distinguish between visual patterns when only one pattern is rewarding [38].

If bees were to prefer the feeder that matched their most recent rewarding food source, they should perform below chance in the sequence experiment (because they would repeatedly visit the last rewarding pattern) but above chance in the positive control experiment (where the same pattern remains rewarding). If, on the other hand, bees were to prefer novelty, they should perform better than chance in the sequence experiment and worse than chance in the positive control experiment.

### 2.6. Measures of Sequence Learning Beyond the First Landing Choice

When the focal bee visited the feeders, it would hover above the platform and visually inspect the two options before choosing one to land on. A well-trained bee would land, walk toward the hole at the center of the overlay, and extend its proboscis to taste the solution. If it landed on the incorrect feeder, upon finding the non-rewarding solution, it would hop off and hover again. Sometimes, even after landing on the correct feeder, the bee would hop off without tasting the contents and inspect the feeders once more.

We recorded the number of these hops (the number of times a bee “explored” before landing to feed and noted the proportion that led to the correct feeder before feeding. We also measured the duration of each feeder visit (trip) for each bee. However, for our sequence learning analysis, we focused on the first landing choice of each visit because it is entirely based on memories acquired on earlier trips.

### 2.7. Statistical Analyses

We collected data from each forager for 125 to 210 min, depending on weather conditions and the bee’s motivation. To ensure equal sample sizes across individuals while still capturing sufficient visits for assessing learning, we restricted our analyses to the first 32 visits per bee. Statistical tests were conducted in R version 3.4.3 [39] and mixed-effects logistic regressions were performed using the “lme4” package [40], unless otherwise specified below. We provide our analysis scripts in the Supplementary Materials.

We tested whether bees performed above random chance by running one-sample *t*-tests on the proportion of correct first landings, comparing each mean against 0.5, the null expectation of random choice. Additionally, we conducted a Fisher’s exact test to compare performances between bees in the sequence experiment and bees in the negative control experiment for visits 17–32, where we expected learning differences to become more pronounced. Paired *t*-tests were then used to compare each bee’s performance in the first half (visits 1–16) versus the second half (visits 17–32) for both the sequence and negative control experiments.

For the sequence experiment, we used JMP Pro v18.0.1 (JMP Statistical Discovery LLC, Cary, NC, USA) to run a General Linear Repeated Measures Mixed Model (GLMM, binomial distribution, logit link) with visit as the repeated measure and bee ID as a random effect because we predicted that the number of correct choices would increase with the number of visits. To test for an affect of aversive solution type (water vs. salt water), we included this treatment type and its interaction with the number of visits in our model. For this experiment, we also used the same software to run a Repeated Measures Mixed Model on the number of times that bees explored before landing to feed (which ranged from 0 to 13 per bee) with bee ID as a random effect and visit as the repeated measure.

We used a mixed-effects logistic regression to determine if the time of day influenced the likelihood of making a correct choice. Each bee made 32 binary (correct vs. incorrect) landing choices, so each bee’s visits were treated as repeated measures.

A power analysis indicated that a sample of 20 bees learning the correct pattern at an asymptotic odds ratio of 2:1 (roughly 0.667 correct choice probability) within 22 visits would provide robust power (β = 0.86) for detecting significant results (α = 0.05) in a similar logistic regression.

We ran linear regressions to determine whether experience affected hopping behavior, using the mean number of hops per visit across bees. Additional linear regressions tested how correct first landings influenced hopping and how visit frequency affected average success, treating each bee as an independent unit (pooling its visits). Further details of our analyses are provided in the Supplementary Materials.

To assess whether the performance of individual bees was better than random, we performed binomial tests on the successes of each bee with a null hypothesis expectation (no learning) of 0.5.

## 3. Results

The bees exposed to neutral solutions (*N* = 14) and aversive solutions (*N* = 6) showed similar performances: there was no significant effect from salt or water treatment (*F*_1,18_ = 0.17, *p* = 0.68) or the interaction visit x treatment (*F*_1,636_ = 0.11, *p* = 0.74), but a significant effect from visit number (*F*_1,637_ = 4.72, *p* = 0.03) because the proportion of correct choices increased with more visits, Figure S1). We therefore pooled the salt and water treatment data in our subsequent analyses. In preliminary assessments, there was no clear difference in how bees behaved upon landing on the feeder blocks, whether they contained water or a saline solution. Previous research suggests that an aversive stimulus may not strengthen learning when a strong reward is already present [30,41], although Avarguès-Weber et al. [27] provide a contrasting perspective when highly concentrated quinine is used as the aversive stimulus.

Bees were able to learn the alternating rewards sequence. They visited the feeder frequently (range: once every 3.9–6.6 min). In the sequence experiment, bees trained to alternating rewards (*N* = 20) had an overall success rate of 0.584, significantly higher than chance (one-sample *t*-test, *t*_19_ = 5.54, *p* < 0.0001). During the first half of their visits, they did not perform better than chance (one-sample *t*-test, *t*_19_ = 1.47, *p* = 0.15). However, they did perform better than chance in the latter half of visitations (visits 17–32, when learning is expected to be greater) with an overall success rate of 0.634 (one-sample *t*-test, *t*_19_ = 5.67, *p* < 0.0001). In our GLMM analysis, there was a significant learning effect over all trials, with correct choices increasing with the number of visits (*F*_1,638_ = 4.73, *p* = 0.03, increasing by 0.2 correct choices per 10 visits).

**Figure 2:**
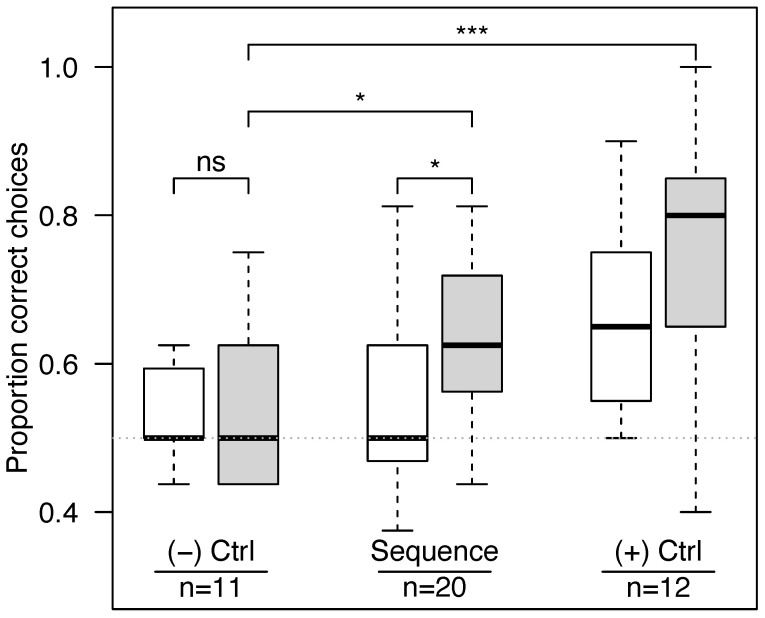
Bees learned in the “sequence” experiment and the positive “(+) Ctrl” experiment, but not in the negative “(−) Ctrl” experiment. White bars represent the first half of each bee’s visits, and gray bars represent the second half (boxplots). The dashed horizontal line represents the 0.5 level expected based on chance alone. The number of trials (each corresponding to a different bee) is shown for each experiment. In the *negative* control experiment, both the rewarding and non-rewarding feeders bore the same pattern, and bees did no better than chance. In the *sequence* experiment, bees faced a challenging task: they had to remember which visual pattern was *not* rewarding on the previous visit, anticipate that it would *become* rewarding on the next visit, and then choose accordingly. They were able to learn this task because they performed significantly better in the second half of their visits. In the *positive* control experiment, one pattern was always paired with a reward while the other was consistently non-rewarding, and bees learned this task with ease, with the highest proportion of correct choices. Horizontal lines show Fisher’s exact test comparisons (“ns” for *p* > 0.05, * *p* < 0.05, and *** *p* < 0.0001).

**Figure 3:**
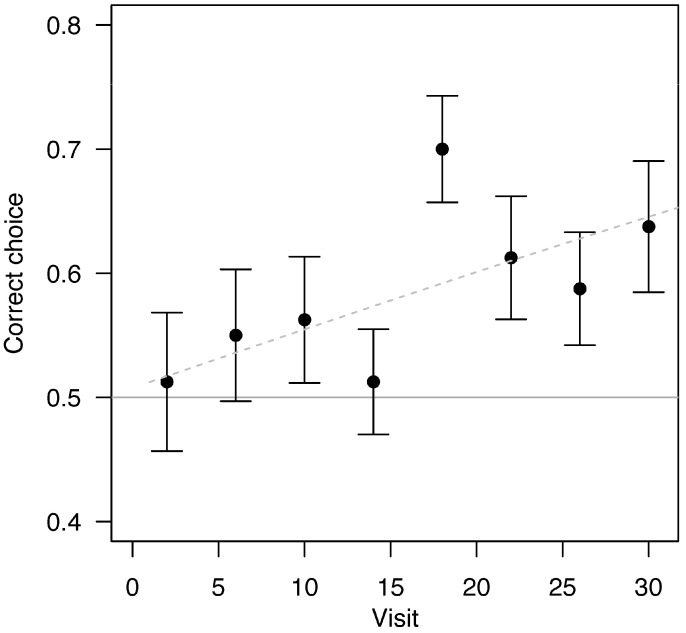
Correct landings increased as the number of visits progressed (*p* = 0.03, increasing by 0.2 correct choices per 10 visits). Each point represents the average of four visits (binned for visualization only; the raw data were not analyzed in bins) for 20 trials, each corresponding to a different bee. The horizontal line indicates the null hypothesis of 50% success, and the dashed trendline shows the logistic regression of correct choices as a function of visit number. Error bars represent standard errors. See Figure S5 for data from the positive and negative control experiments.

In the latter half of visitations, bees performed better in the sequence learning experiment than in the negative control experiment (Fisher’s exact test, success rates of 0.634 versus 0.539 respectively, *p* = 0.044, Figure 2). Bees in the sequence experiment improved at landing on the correct feeder with successive visits (Figure 3 and Figure S2, logistic regression with success as a function of visit numbers and bee as a repeated measure, *z* = 2.17, *p* = 0.030).

In the negative control experiment, bees trained to two identical patterns (*N* = 11) had an overall success rate of 0.534, not significantly different from chance (one-sample *t*-test, *t*_10_ = 1.75, *p* = 0.11). In the positive control experiment, bees trained to constant rewards (*N* = 12) had an overall success rate of 0.704, significantly higher than chance (one-sample *t*-test, *t*_11_ = 6.01, *p* < 0.0001) and much higher than the negative control in the second half of their visits (Fisher’s exact test, *p* < 0.001).

Most bees were quite active and, after their first landing on either feeder, would often fly off and land again on the same feeder or the other feeder before imbibing from the rewarding feeder. Per bee, the average number of landings before imbibing ranged from 1.65 to 3.40 for the bees in the sequence experiment. With experience (visit number), bees did not significantly change the number of explorations between feeders before they landed to feed (*F*_1,619_ = 2.17, *p* = 0.14).

**Figure 4:**
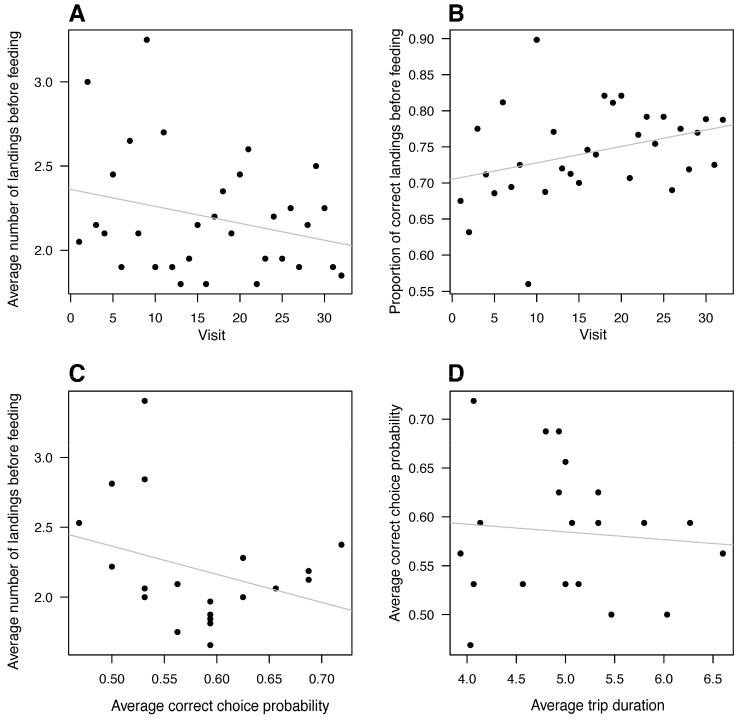
Bee landing behavior did not change significantly with experience. (**A**) The average number of landings made before feeding did not correlate with the number of visits. (**B**) The proportion of correct landings (ratio of landings) showed no correlation with the number of visits. (**C**) There was no relationship between the correctness of the first landing choice and the number of landings before feeding, and (**D**) average trip duration showed no relationship with the correctness of the first landing choice. Each point represents a different bee. The solid lines are the linear regression lines.

There was no correlation between visit number and how many times a bee flew off and landed again before drinking (Figure 4A, linear regression, *F*_1,30_ = 2.28, *p* = 0.141). There was also no significant improvement in the ratio of landings on the correct feeder as a function of visit number (Figure 4B, linear regression, *F*_1,30_ = 3.74, *p* = 0.063). The average first-landing success rate of each bee did not correlate with how many times (on average) it landed on and jumped between the feeders before being rewarded (Figure 4C, linear regression, *F*_1,18_ = 2.07, *p* = 0.168). There was no correlation between average trip duration and average correctness per bee (Figure 4D, linear regression, *F*_1,18_= 0.144, *p* = 0.709).

As expected, individual bees showed different learning curves (Figures S1 and S3), and some were stronger learners (range: 23–15 correct choices in 32 visits, with binomial *p* values from 0.02–1). Bees that failed to learn the sequence either landed randomly on the feeders or developed a bias to always land on the same pattern first, then hop to the other feeder if the first contained no reward (Figure S4 shows the pattern each bee first landed on for each visit). Alternatively, they may have followed a “choose what I chose last” strategy but with some error.

## 4. Discussion

Our sequence task likely posed a significant challenge for the bees because of memory interference. Neither pattern remained consistently rewarding, forcing bees to infer that the previously non-rewarding pattern would become rewarding on the next trip. They thus needed to override their prior association of a specific visual pattern with the *absence* of a reward and instead recognize it as the new rewarding option on the subsequent visit. Cheng and Wignall [42] have shown that honey bees tend to retain memories and encounter difficulties when replacing old memories with new ones. Nonetheless, our results show that bees were able to learn the alternating sequence task after 16 visits, although the effect size was modest (the odds ratio of correct to incorrect first landings for visits 25–32 was 1.58). Comparable binomial choice experiments likewise report steady increases in success at the population level but substantial individual variability within 30 trials [20,43,44].

Our work differs from reversal learning [45], which examines how animals adapt to stable but changing reward contingencies when a previously non-rewarding stimulus becomes rewarding after a set reversal point. Instead, we required bees to follow a dynamic alternation of rewards, remembering which pattern had last been rewarding and predicting that the previously unrewarding pattern would become rewarding on the next visit. In reversal learning, the rule stabilizes after the reversal, whereas our experiment demanded that bees continuously recall the last outcome and apply this knowledge to the next trip. As might be expected, we found considerable variation in the ability of individuals to perform this difficult task, consistent with findings by Finke et al. [45,46].

It also seems unlikely that bees succeeded by detecting odor differences between sucrose and water feeders, or by following scent marks. Our negative control experiment showed no evidence of learning without a distinctive pattern sequence. Moreover, if a reliable odor cue had been available, we would expect much better than 58% accuracy in the sequence experiment, based on the literature [47,48].

Many of our foragers failed to learn the sequence (Figure S3), perhaps because the cost of making a wrong decision was low. The artificial flowers in our experiment were separated by just 6 cm, a trivial distance in terms of both time and energy for the bee. We chose this short distance to ensure that bees would return to the choice array. Preliminary trials with larger separations (10–15 m) led bees to abandon the feeders and forage elsewhere. Consequently, it may not be surprising that, in a relatively difficult sequence task, bees performed only slightly above chance (mean of 58% correct choices) while performing substantially better (70%) in the positive control experiment.

Although our setup was controlled and somewhat artificial, sequence learning remains ecologically relevant for bees. In nature, they must track fluctuating nectar rewards influenced by competition, floral replenishment, and environmental shifts [16,22]. Rather than relying solely on immediate cues, bees can benefit from predicting nectar availability [17]. This predictive skill also underlies bumblebee traplining, where bees form efficient visitation routes based on past experiences [26]. Despite requiring extensive training and achieving moderate success in our trials, bees may perform better in the wild because additional sensory cues could guide their sequential foraging.

Other factors may also explain why many bees did not master the alternating sequence. Some bees may have learned the correct rule, “the pattern that rewarded me last time will not reward me this time”, yet lacked a strong capacity to remember which pattern they last visited because both patterns had been rewarded equally over time. They might have confused the two patterns or simply failed to discriminate them consistently. Alternatively, individual behavioral preferences could play a role, such that some scouts or “win-shift” bees had a strong drive to explore novel sources and might have scored well without truly learning the rule. However, we find significant learning overall, despite the complexity of this task.

In the sequence learning experiment, we pseudorandomly changed the feeder’s location on the platform to reduce the influence of visual landmarks on landing behavior [49]. This step may have complicated the bees’ task by increasing uncertainty, which often impairs learning in many animals [50]. Not surprisingly, bees in the positive control experiment exhibited a higher mean success rate (70%) than those in the sequence experiment (58%), yet even this was lower than the 85–90% typical in simpler discrimination tasks where a single choice is consistently rewarded [47,48]. Future studies might test whether maintaining context embedding improves sequence learning, given that standard protocols against site bias often involve swapping feeders or visual stimuli after each visit [51].

We observed that bees failing to learn the sequence either continued to land randomly or developed a preference for initially landing on a particular pattern. Even for bees that did not learn the correct sequence, a lack of reward on their first landing continued to provoke exploratory behavior. This exploratory behavior also persisted in bees that successfully learned the sequence. This result reinforces findings in ants, where many individuals faced with an insoluble task began favoring one side of the Y-maze or turning in one direction [52,53].

Honey bees typically prefer a win-shift strategy—avoiding recently emptied flowers—but can learn win-stay when that rule yields higher returns [37]. A strict win-shift approach might have allowed bees to excel in our alternation task, yet, in examining individual behaviors, only bee #1 (Figure S3) performed near flawlessly, and then only after 22 visits. Additionally, bees strongly favored win-stay in the positive control experiment, confirming that they tend to persist with a recently rewarded site and underscoring why the alternating sequence task was more difficult. Although bumblebees have been reported to adopt a win-stay/lose-switch strategy after about 20 bouts of training [54], our honey bees did not consistently show such a pattern here.

Inter-individual variability in honey bee learning and foraging strategies is well established [55]. Relevantly, Finke et al. [45] showed that some honey bee foragers excel in reversal learning while others struggle to override prior associations. Our data are insufficient for a clear categorization of bees based on their foraging choices, but some bees may have relied on a win-stay strategy—returning to previously rewarding feeders rather than adopting the alternating sequence rule—while others attempted spatial heuristics, focusing on feeder position rather than visual patterns despite randomized placement. Variability in honey bee associative learning and memory retention has been noted in both olfactory and visual tasks, with certain bees demonstrating faster acquisition and higher retention [56,57]. We also considered whether bees might have relied on spatial location rather than visual pattern cues in terms of individual strategies. However, because feeder locations were randomized and carefully monitored, we detected no systematic bias in feeder choice. The absence of a consistent error pattern suggests that visual patterns were the primary cue.

Our findings add to existing evidence that honey bees can learn sequences under various conditions. Collett et al. [25] showed that bees trained on visual patterns could learn to anticipate specific stimuli at particular points along a route. Menzel [58] similarly noted that landmarks closest to the decision point exert the strongest influence on bees’ navigation, while more distant landmarks have a diminishing effect. Mota and Giurfa [59] demonstrated that repeated olfactory reversal learning eventually compromised bees’ ability to update their responses after enough reversals. Against this backdrop, our alternating design required bees to reassess the reward cue on every single visit, and they still achieved a modest but meaningful level of success.

Research in humans further underscores the complexity of learning sequences. Even when people fail to grasp the abstract rules governing stimulus transitions, they still develop correct statistical biases toward frequent transitions [60]. In these experiments, participants relied on associative mechanisms to detect embedded relationships, a process that may also operate in bees. Honey bees could similarly acquire implicit knowledge of a reward sequence by incrementally associating specific cues with subsequent outcomes, even if they do not explicitly “comprehend” the rule itself.

Statistical learning itself encompasses many forms, and no unified mechanism explains how different species master stimulus patterns or behaviors [61]. Nonetheless, the neural architecture of insects supports the plausibility of this type of learning [62,63]. Our work strengthens this view by demonstrating that honey bees can learn a visual sequence with reward intervals that exceed a few seconds. This ability is ecologically relevant to foraging and reaffirms the value of honey bees as a model for studying animal cognition [64,65].

## Data Availability

On publication, all data will be made freely available at Zenodo.org at DOI: 10.5281/zenodo.14589949.

## Supplementary Materials

The following supporting information can be downloaded at: https://www.mdpi.com/article/10.3390/insects16040358/s1, Figure S1. Success rates for each bee in the sequence experiment; Figure S2. Overall learning in the population; Figure S3. Performance of individual bees in the sequence experiment; Figure S4. Landing choices of individual bees in the sequence experiment; Figure S5. As bees gained more experience, they made correct landings more often; R code; JMP code.
