# Supplementary material for: Honey Bees Can Use Sequence Learning to Predict Rewards from a Prior Unrewarded Visual Stimulus
Source: Insects. 2025 Mar 31;16(4):358. doi: 10.3390/insects16040358 (PMC12027691; doi:10.3390/insects16040358)
Supplement: Supplementary file 1 [file insects-16-00358-s001.zip › insects-3449488-supplementary.pdf]

insects

MDPI

SUPPLEMENTARY MATERIAL

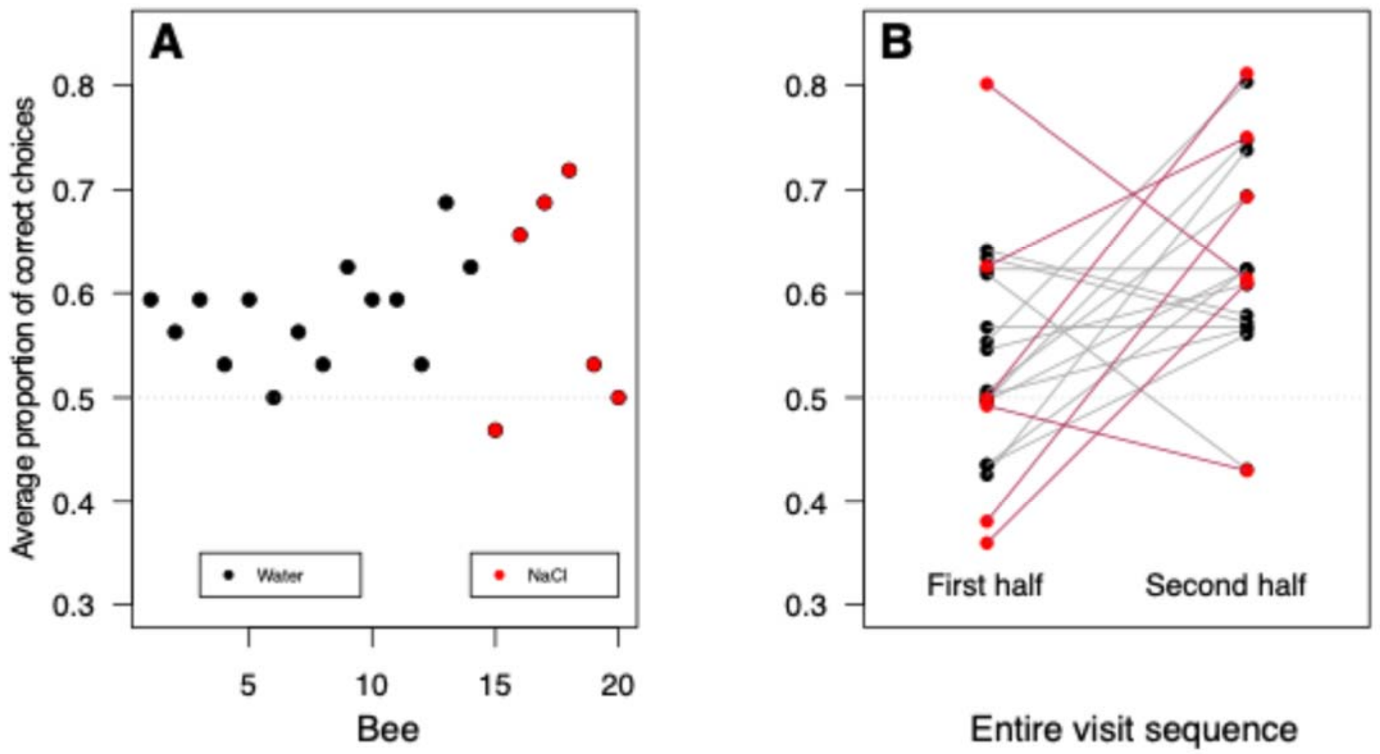

Figure S1. Success rates for each bee in the sequence experiment. Each dot represents the proportion of correct first landings for each bee over its 32 visits. The dotted grey line shows the average chance expectation of 50% success. The black dots represent bees that received distilled water as their non-rewarding solution, whereas the red dots represent bees that received 3M NaCl as their non-rewarding solution. A) The average success of each bee over 32 visits is shown. B) Each bee's performance during the first half and the second half of the visits is shown. Bees performed better in the second half of the visits than in the first half (paired t-test, t_{19} = -2.79, P = 0.012), irrespective of the type of neutral or aversive solution in the non-reward feeder (no significant effects of salt or water treatment ($F_{1,18}$=0.17, P = 0.68). However, we acknowledge that the number of NaCl-exposed bees was small.

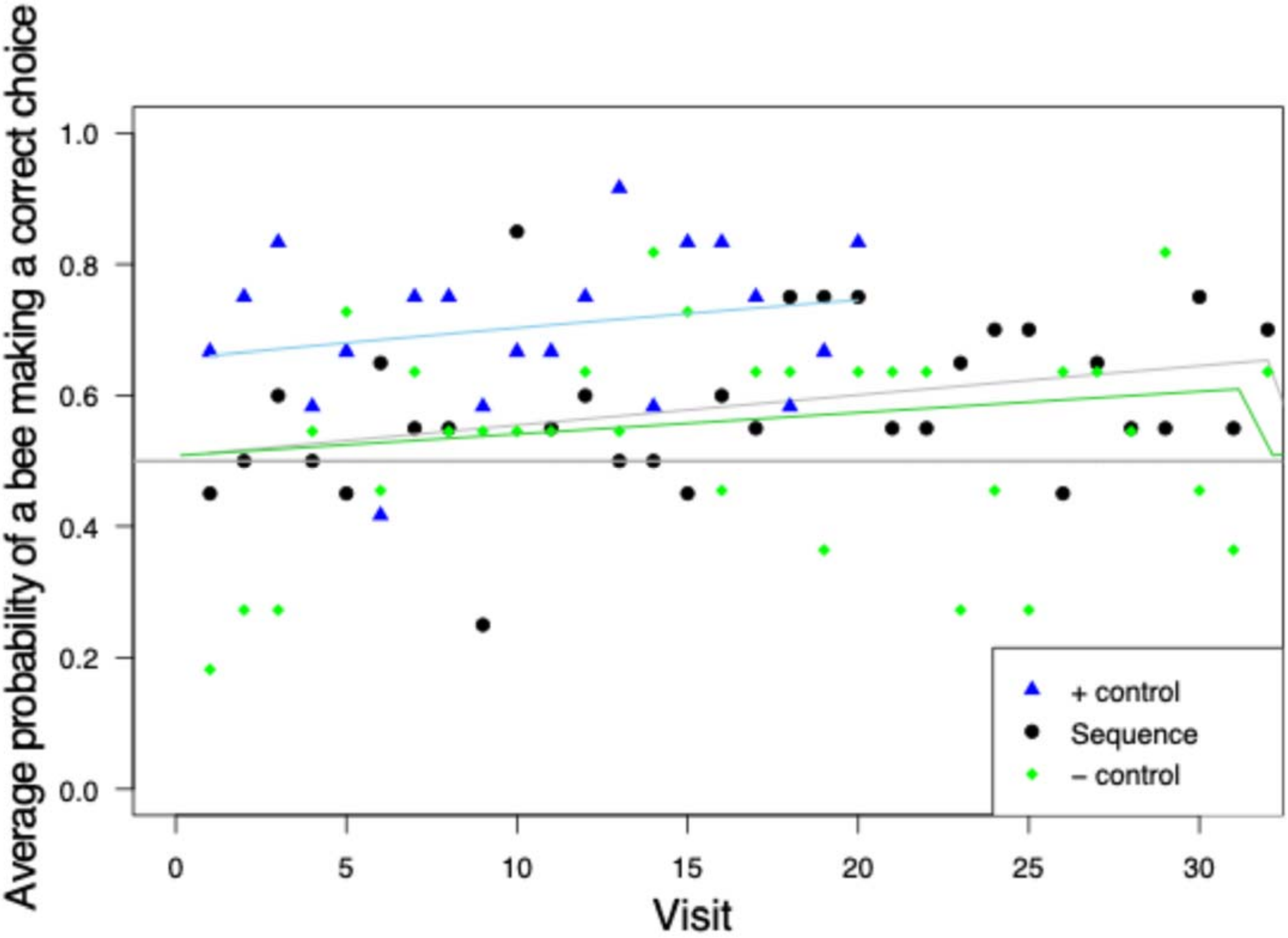

Figure S2. Overall learning in the population**.** Correct choices increased significantly as the bees continued visiting the feeders. The grey solid trendline is based on a logistic regression of success as a function of visit number, with repeated measures for each bee (z = 2.17, P = 0.030). The dashed grey line shows the average chance expectation of 50% success.

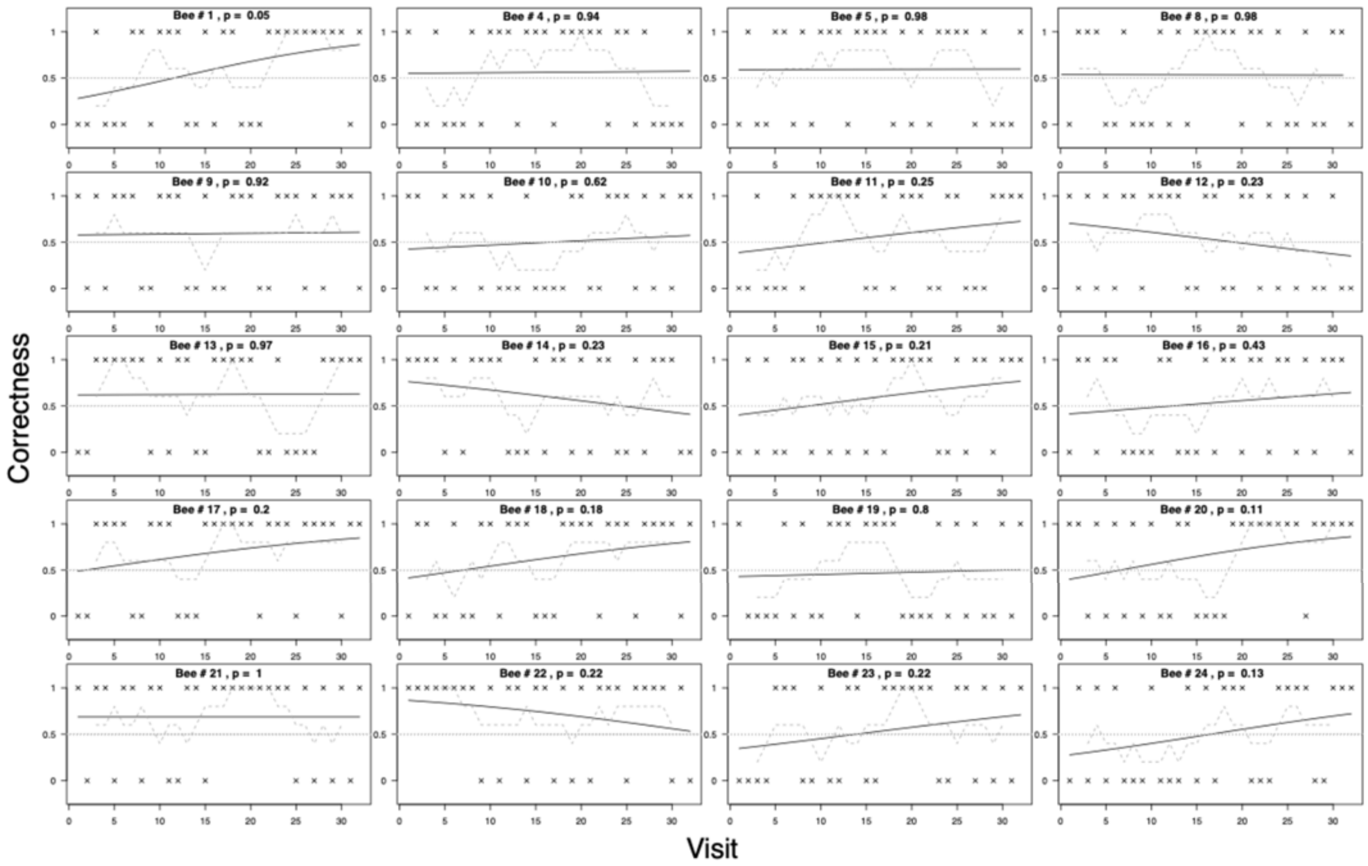

Figure S3. Performance of individual bees in the sequence experiment. Each panel depicts the choices of a single bee. Bee identity and the value for the logistic regression of correct choices versus visit number appear at the top of each panel. The black solid trendline represents the logistic regression, and the horizontal grey dotted line shows the null hypothesis expectation of 50% correctness. Bees 2, 3, 6, and 7 did not complete more than 30 visits and were therefore excluded from our analyses requiring at least 32 visits per bee.

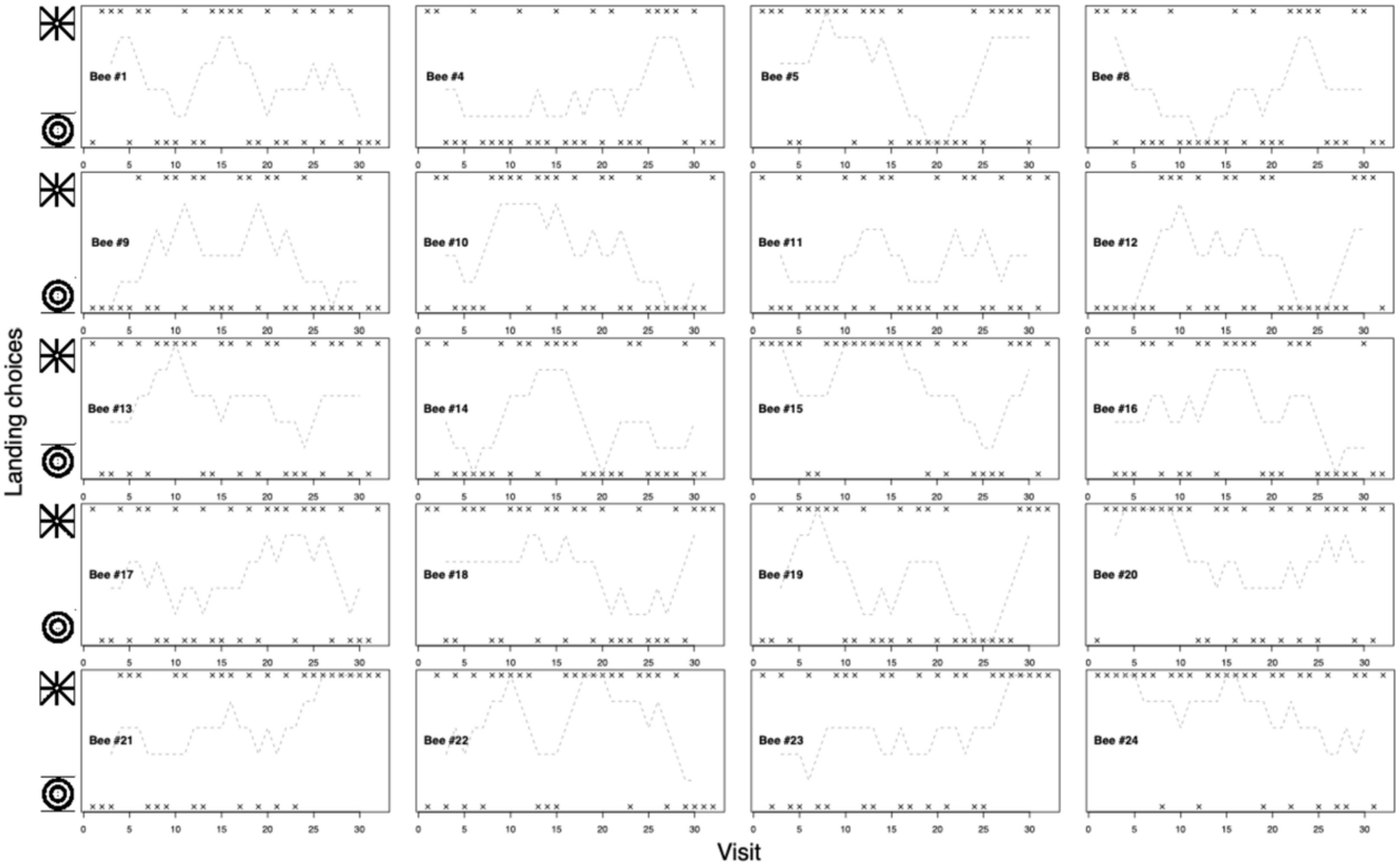

Figure S4. Landing choices of individual bees in the sequence experiment. The grey trendlines are a moving-window average of correct choices in 5 visits (2 before to 2 after). Bees 5, 9, 10, 12, 16, 19, 21, and 23 may have developed a tendency to stick to a single pattern in the later stages of the experiment. This behavior resulted in lower performance due to the alternating reward structure.

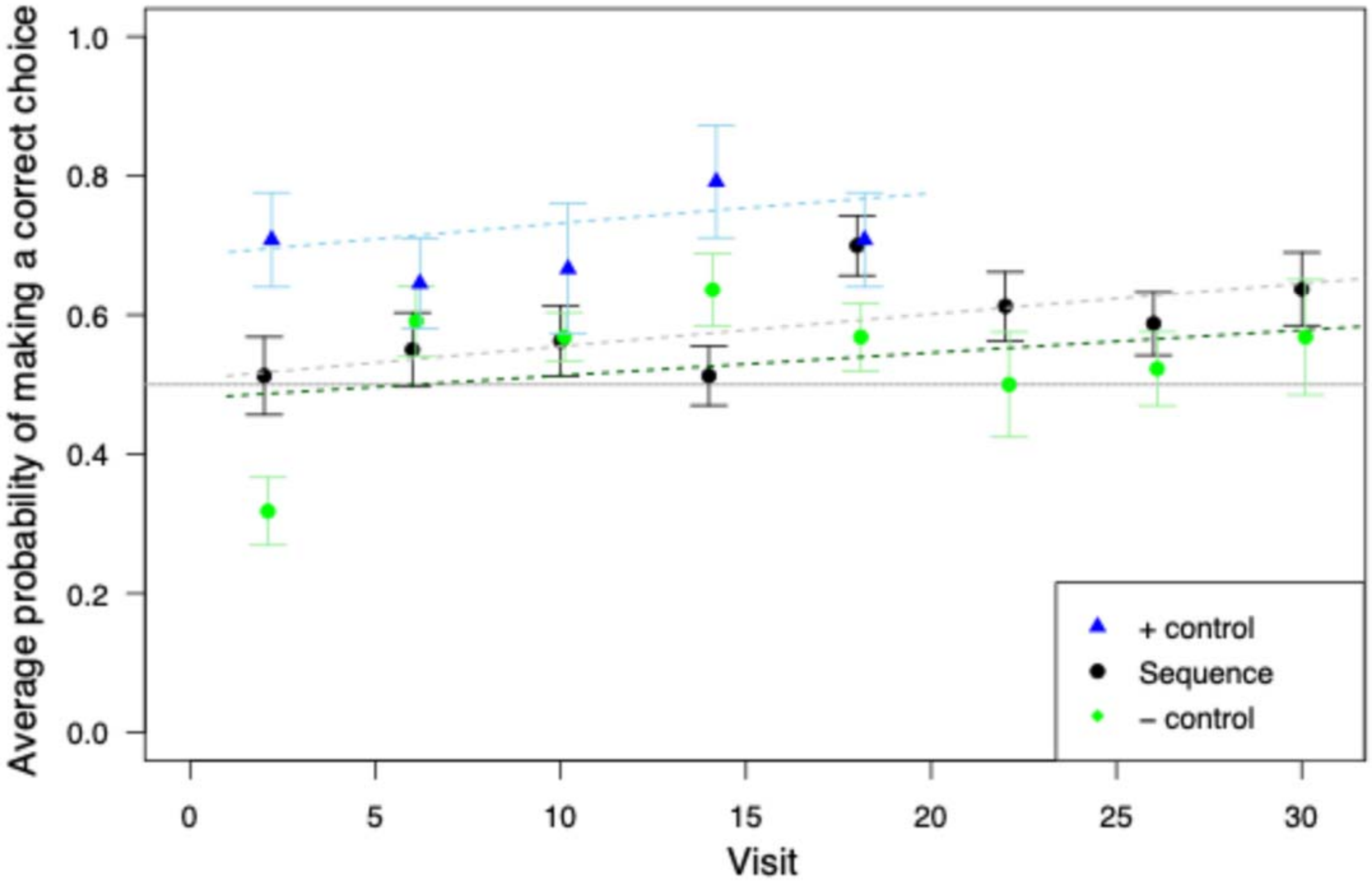

Figure S5. As bees gained more experience, they made correct landings more often. Each point shows the average of four visits, each from a different bee. Note that this is only visualization, the data were not analyzed in bins. The horizontal grey line represents the 50% success expected by chance, and the dashed trendlines depict the logistic regression of correct choices as a function of visit number in each experiment. Standard errors are shown. The effect of visit number on correct choices was significant in the sequence experiment (logistic regression with success as a function of visit number and bee as a repeated measure, $z = 2.17$, $P = 0.030$) but not in the positive control experiment ($z = 0.951$, $P = 0.34$) or the negative control experiments ($z = 1.13$, $P = 0.26$). In the positive control experiment, the intercept was significantly higher than 0.5 ($z = 2.17$, $P = 0.03$), suggesting very rapid learning of the constant reward rule. In the negative control experiment, the intercept was indistinguishable from 0.5 ($z = -0.37$, $P = 0.72$), indicating that bees could not find the rewarding feeder without reliable visual cues. The intercept was also not different from 0.5 in the sequence experiment at the outset ($z = 0.19$, $P = 0.85$).

R code for sequence analyses:

```
#require(ggplot2)
#require(GGally)
#require(reshape2)
library(lme4)
#require(compiler)
#require(parallel)
#require(boot)
#require(lattice)

#Input data and separate treatments
sequencesall=read.csv(file = 'C:/Users/bahra/Desktop/Dataset_sequence_learning_DOUBLECHECKED.csv',header=T)
seql=subset(sequencesall,sequencesall$experiment=='seq')
negseql=subset(sequencesall,sequencesall$experiment=='neg')
posseql=subset(sequencesall,sequencesall$experiment=='pos')

#logistic regression
seqlo=seql ; seqlo$Bee=as.factor(seqlo$Bee) #Setting bee as a factor
summary(glmer(Correct ~ Visit + (Visit|Bee), family=binomial,data=seqlo))#p=0.078 marginally significant
negseqlo=negseql; negseqlo$Bee=as.factor(negseqlo$Bee) #Setting bee as a factor
summary(glmer(Correct ~ Visit + (Visit|Bee), family=binomial,data=negseqlo)) #p=0.25 negative control bees don't learn
posseqlo=posseql; posseqlo$Bee=as.factor(posseqlo$Bee) #Setting bee as a factor
summary(glmer(Correct ~ Visit + (Visit|Bee), family=binomial,data=posseqlo)) #p=0.25 negative control bees don't learn

# Negative Control experiment: is it different from sequence experiment?
# on the entire data set: fisher's test p-value = 0.14
tc= sum(seql$Correct)
ti = nrow(seql)-tc
cc= sum(negseql$Correct)
ci= nrow(negseql)-cc
ratios = rbind(c(tc,ti),c(cc,ci))
fisher.test(ratios)
#only the second half: p-value = 0.044 this is where learning has had time to occur
seqlu=subset(seql,seql$Visit>16)
negseqlu=subset(negseql,negseql$Visit>16)
tc= sum(seqlu$Correct)
ti = nrow(seqlu)-tc
cc= sum(negseqlu$Correct)
ci= nrow(negseqlu)-cc
ratios = rbind(c(tc,ti),c(cc,ci))
fisher.test(ratios)
#only the second half: p-value = 0.044 this is where learning has had time to occur
posseqlu=subset(posseql,posseql$Visit>10)
negseqlu=subset(negseql,negseql$Visit>16)
tc= sum(posseqlu$Correct)
ti = nrow(posseqlu)-tc
cc= sum(negseqlu$Correct)
ci= nrow(negseqlu)-cc
ratios = rbind(c(tc,ti),c(cc,ci))
fisher.test(ratios)
```

```
##...Fig 2...## Distribution of success in bees
#All visits
bees = unique(seql$Bee)
listper=NULL
lineslope=NULL
for(o in bees) {
beet=subset(seql,seql$Bee==o )
beet=subset(beet, beet$Visit <33)
mus=(sum(beet$Correct)/32)
listper=c(listper,mus)}
t.test(listper-0.5) #one-sample t test to see if average is 0.5
bees = unique(negseql$Bee)
listpern=NULL
for(o in bees) {
beet=subset(negseql,negseql$Bee==o )
beet=subset(beet, beet$Visit <33)
mus=(sum(beet$Correct)/32)
listpern=c(listpern,mus)}
t.test(listpern-0.5) #one-sample t test to see if average is 0.5
bees = unique(posseql$Bee)
listperp=NULL
for(o in bees) {
beet=subset(posseql,posseql$Bee==o )
beet=subset(beet, beet$Visit <33)
mus=(sum(beet$Correct)/20)
listperp=c(listperp,mus)}
t.test(listperp-0.5) #one-sample t test to see if average is 0.5
##first half of visits
#seql=simul #if you want to compare the power analysis results based on an artificial sample, you should erase the # from the beginning of this line after running the sampling model.Refer to line 386 for more details on this simulation sample.
bees = unique(seql$Bee)
listper1=NULL
for(o in bees) {
beet=subset(seql,seql$Bee==o )
beet=subset(beet, beet$Visit <17)
mus=(sum(beet$Correct)/16)
listper1=c(listper1,mus)}
t.test(listper1-0.5)
bees = unique(negseql$Bee)
listpern1=NULL
for(o in bees) {
beet=subset(negseql,negseql$Bee==o )
beet=subset(beet, beet$Visit <17)
mus=(sum(beet$Correct)/16)
listpern1=c(listpern1,mus)}
t.test(listpern1-0.5)
bees = unique(posseql$Bee)
listperp1=NULL
for(o in bees) {
beet=subset(posseql,posseql$Bee==o )
```

```
beet=subset(beet, beet$Visit <11)
mus=(sum(beet$Correct)/10)
listperp1=c(listperp1,mus)}
t.test(listperp1-0.5)
####second half of visits
bees = unique(seql$Bee)
listper2=NULL
for(o in bees) {
beet=subset(seql,seql$Bee==o )
beet=subset(beet, beet$Visit >16)
mus=(sum(beet$Correct)/16)
listper2=c(listper2,mus)}
t.test(listper2-0.5)
bees = unique(negseql$Bee)
listpern2=NULL
for(o in bees) {
beet=subset(negseql,negseql$Bee==o )
beet=subset(beet, beet$Visit >16)
mus=(sum(beet$Correct)/16)
listpern2=c(listpern2,mus)}
t.test(listpern2-0.5)
bees = unique(posseql$Bee)
listperp2=NULL
for(o in bees) {
beet=subset(posseql,posseql$Bee==o )
beet=subset(beet, beet$Visit >10)
mus=(sum(beet$Correct)/10)
listperp2=c(listperp2,mus)}
t.test(listperp2-0.5)
par(mfrow=c(1,1))
collist=c('white','lightgrey','dimgrey','white','lightgrey','dimgrey','white','lightgrey','dimgrey')
boxplot(listpern1,listpern2,5,listper1,listper2,5,listperp1,listperp2,col=collist,ylim=c(0.3,1.05),ylab='Proportion correct choices',las=1)
abline(h=0.5,col='darkgrey',lty=3)
segments(1,0.85,2,0.85);segments(2,0.94,5,0.94);segments(2,1.03,8,1.03);segments(4,0.85,5,0.85)
segments(0.75,0.32,2.25,0.32);segments(3.75,0.32,5.25,0.32);segments(6.75,0.32,8.25,0.32)
segments(1,0.85,1,0.83);segments(2,0.85,2,0.83);segments(2,0.94,2,0.92);segments(5,0.94,5,0.92)
segments(4,0.85,4,0.83);segments(5,0.85,5,0.83);segments(2,1.03,2,1.01);segments(8,1.03,8,1.01)
text(1.5,0.345,"(-) Ctrl");text(4.5,0.345,"Sequence");text(7.5,0.345,"(+) Ctrl")
text(1.5,0.30,"n=11");text(4.5,0.30,"n=20");text(7.5,0.30,"n=12")
text(4.5,0.87,"*");text(3.5,0.96,"*");text(5,1.05,"***");text(1.5,0.87,'ns')
t.test(listper1,listper2,paired=T)
t.test(listpern1,listpern2,paired=T)
t.test(listperp2,listpern2)

## ...Fig 3...## Logistic regression shown for bins of 4-visit periods for ease of visualization
par(mfrow=c(1,1))
tallys=NULL
seq=seql
bees = unique(seql$Bee)
plot(0,0, las=1,xlim=c(0,30.5),ylim=c(0.0,1),col='white',xlab='Visit',ylab='Correct choice')
abline(h=0.5,col='darkgrey',lty=1)
```

```
tally = NULL
for (o in 0:7) {
p=subset(seq,seq$Visit %in% c((o*4+1):(4*(o+1))))
suc=sum(p$Correct)
###calculate standard deviation/error and add error bars
tri=nrow(p)
SDB=NULL
for (i in bees) {
beeg=subset(p,p$Bee==i)
h=mean(beeg$Correct)
SDB=c(SDB,h)}
sdblock=sd(SDB)/sqrt(20)
arrows((o*4)+2,suc/tri-sdblock,(o*4)+2,suc/tri+sdblock,col='black',code=3,angle=90,length=0.1)
points((o*4)+2,suc/tri,pch=19,col='black')
tally = c(tally,(suc/tri))}
modelallvis=glm(Correct ~ Visit + (Visit|Bee), family=binomial,data=seql)
pred= predict(modelallvis,x=seql$Visit,type="response")
lines(pred,col='grey',xlim=c(0,32),lty='dashed')
#negative control
tallys=c(tallys,tally)
seq=negseql
bees = unique(seq$Bee)
tally = NULL
for (o in 0:7) {
p=subset(seq,seq$Visit %in% c((o*4+1):(4*(o+1))))
suc=sum(p$Correct)
###calculate standard deviation/error and add error bars
tri=nrow(p)
SDB=NULL
for (i in bees) {
beeg=subset(p,p$Bee==i)
h=mean(beeg$Correct)
SDB=c(SDB,h)}
sdblock=sd(SDB)/sqrt(11)
arrows((o*4)+2.1,suc/tri-sdblock,(o*4)+2.1,suc/tri+sdblock,col='lightgreen',code=3,angle=90,length=0.1)
points((o*4)+2.1,suc/tri,pch=16,col='green')
tally = c(tally,(suc/tri))}
modelallvis=glm(Correct ~ Visit + (Visit|Bee), family=binomial,data=negseql)
pred= predict(modelallvis,x=seql$Visit,type="response")
lines(pred,col='darkgreen',xlim=c(0,32),lty='dashed')
#positive control
tallys=c(tallys,tally)
seq=posseql
bees = unique(seq$Bee)
tally = NULL
for (o in 0:4) {
p=subset(seq,seq$Visit %in% c((o*4+1):(4*(o+1))))
suc=sum(p$Correct)
###calculate standard deviation/error and add error bars
tri=nrow(p)
SDB=NULL
for (i in bees) {
```

```
beeg=subset(p,p$Bee==i)
h=mean(beeg$Correct)
SDB=c(SDB,h)}
sdblock=sd(SDB)/sqrt(12)
#print(binom.test(suc,tri))
arrows((o*4)+2.2,suc/tri-sdblock,(o*4)+2.2,suc/tri+sdblock,col='skyblue',code=3,angle=90,length=0.1)
points((o*4)+2.2,suc/tri,pch=16,col='blue')
tally = c(tally,(suc/tri))}
abline(h=0.5,col='darkgrey',lty=3)
modelallvis=glm(Correct ~ Visit + (Visit|Bee), family=binomial,data=posseql)
pred= predict(modelallvis,x=seql$Visit,type="response")
lines(pred,col='skyblue',lty='dashed') #the extra line from visit 20 onward is an artefact of the "predict" functionthat loops it around again
tallys=c(tallys,tally)
text(23,0.3,'Sequence');points(pch=19,col='black',20,0.3)
text(23,0.20,'+ control');points(pch=19,col='blue',20,0.2)
text(23,0.1,'- control');points(pch=19,col='green',20,0.1)

##...Fig 4...##Other measures of learning:
par(mfrow=c(2,2))
# 4A-Do bees jump around less before drinking as they become more experienced? Not significantly (p=0.14).
conf_av=NULL
for (i in 1:32) {
vis=subset(seql,seql$Visit==i)
av=mean(vis$Explor_pre_feed)+1
conf_av=rbind(conf_av,cbind(i,av))}
plot(conf_av[,1],(conf_av[,2]),pch=16,ylab='Average landings before feeding',xlab='Visit',las=1)
linm=lm((conf_av[,2])~conf_av[,1])
abline(linm,col='gray')
summary(linm)

# 4B- Do they become less uncertain (land on the correct one more)? marginally significantly (p=0.063).
conf_av=NULL
for (i in 1:32) {
vis=subset(seql,seql$Visit==i)
av=mean((vis$Explor_pre_feed-vis$Explor_cor)/(vis$Explor_pre_feed+1))
conf_av=rbind(conf_av,cbind(i,av))}
plot(conf_av[,1],1-(conf_av[,2]),pch=16,ylab='Ratio landings on correct pattern before feeding',xlab='Visit',las=1)
linm=lm(1-(conf_av[,2])~conf_av[,1])
abline(linm,col='gray')
summary(linm)

# 4C- Are bees who land on fewer feeders the more correct ones? No (p=0.17).
confusion=NULL
percent=NULL
bees=unique(seql$Bee)
for(i in bees){
bee=subset(seql,seql$Bee==i)
confusion=c( confusion,mean(bee$Explor_pre_feed+1,na.omit=1))
percent=c(percent,(sum(bee$Correct)/32))
}
plot(percent,confusion,pch=16,xlab='Average correct choice probability',ylab='Average landings before feeding',las=1)
```

```
lin=lm(confusion~percent)
abline(lin,col='gray')
summary(lin)

# 4D- Return time depending on learning? No (p=0.70).
beetimes=NULL
pvallist=NULL
corres=NULL
for (i in bees) {
beed=subset(seql,seql$Bee==i)
bt=beed$Time
bth=floor(bt/100)
btm=bt%%100
btf=(bth*60)+btm-(bth[1]*60)-(btm[1]+1)
beetimes=c(beetimes,(btf[30]/30))
corre=sum(beed$Correct)
example=glm(beed$Correct~beed$Visit, family=binomial)
pvals=summary(example)
pval=coefficients(pvals)[8]
pvallist=c(pvallist,pval)
corres=c(corres,corre)
}
plot(beetimes, corres/32,pch=16,ylab='Average correct choice probability',xlab='Average trip duration',las=1)
corresr=corres/32
linm=lm(corresr~beetimes)
abline(linm,col='gray')
summary(linm)

##
##
## Supplementary material

##...Fig S1...##
par(mfrow=c(1,2))
plot(listper,ylim=c(0.3,0.85),pch=19,las=1,ylab='Average correct choices',xlab='Bee')
points(c(15:20),listper[15:20],col='red',pch=16)
abline(h=0.5,col='darkgrey',lty=3)
legend(pch=19,col=1,3,0.35,'Water');legend(pch=19,col='red',14,0.35,'NaCl')

rands=runif(20,-0.02,0.02) #this line is just to avoid overlapping in plotting, the data is not changed
bees = unique(seql$Bee)
plot(0,0,xlim=c(0.6,2.4),ylim=c(0.3,0.85),col='white',las=1);abline(h=0.5,lty=3,col="grey")
for(i in 1:14) {
points(1,listper1[i]+rands[i],col=1,pch=16)
points(2,listper2[i]+rands[i],col=1,pch=16)
segments(1,listper1[i]+rands[i],2,listper2[i]+rands[i],col='darkgrey',lwd=1)
}
for(i in 15:20) {
points(1,listper1[i]+rands[i],col='red',pch=16)
points(2,listper2[i]+rands[i],col='red',pch=16)
segments(1,listper1[i]+rands[i],2,listper2[i]+rands[i],col='maroon',lwd=1)
}
```

```
text(1.5,0.32,'First halfSecond half')

##...Fig S2...## Average correct choice per visit (not all visits have same replicate number)
{par(mfrow=c(1,1))
seqavs=NULL
seq_av= NULL
for (i in 1:32) {
seq_vis=subset(seql,seql$Visit==i)
av=mean(seq_vis$Correct)
seq_av=rbind(seq_av,cbind(i,av))}
plot(seq_av[,1],seq_av[,2],las=1,ylim=c(0,1),xlim=c(0,31.2),pch=19,xlab='Visit',ylab="Average correct choices probability")
abline(h=0.5,col='darkgrey',lty=3)
modelallvis=glm(Correct ~ Visit + (Visit|Bee), family=binomial,data=seql)
pred= predict(modelallvis,x=seql$Visit,type="response")
lines(pred,col='grey')
seqavs=rbind(seqavs,seq_av)
seq_av= NULL
for (i in 1:32) {
seq_vis=subset(negseql,negseql$Visit==i)
av=mean(seq_vis$Correct)
seq_av=rbind(seq_av,cbind(i,av))}
points(seq_av[,1],seq_av[,2],col='green',las=1,ylim=c(0,1),pch=18,xlab='Visit',main='Probability of Correct Sequence Choice',ylab="Average correct choices probability")
abline(h=0.5,col='darkgrey')
modelallvis=glm(Correct ~ Visit + (Visit|Bee), family=binomial,data=negseql)
pred= predict(modelallvis,x=seql$Visit,type="response")
lines(pred,col='limegreen')
seqavs=rbind(seqavs,seq_av)
seq_av= NULL
for (i in 1:20) {
seq_vis=subset(posseql,posseql$Visit==i)
av=mean(seq_vis$Correct)
seq_av=rbind(seq_av,cbind(i,av))}
points(seq_av[,1],seq_av[,2],col='blue',las=1,ylim=c(0,1),pch=17,xlab='Visit',main='Probability of Correct Sequence Choice',ylab="Average correct choices probability")
abline(h=0.5,col='darkgrey')
modelallvis=glm(Correct ~ Visit + (Visit|Bee), family=binomial,data=posseql)
pred= predict(modelallvis,x=seql$Visit,type="response")
lines(pred,col='skyblue') #Repeats the predicted trendline after visit 21, but it does not mean anything.
legend(pch=18,col='green',27,0.15,'- control')
legend(pch=17,col='blue',27,0.35,'+ control')
legend(pch=19,col='black',27,0.25,'Sequence')
seqavs=rbind(seqavs,seq_av)
seqavs=cbind(seqavs,c(rep(1,32),rep(2,32),rep(3,20)))
}
##...Fig S3..## Individual bees' logistic regression:
bees = unique(seql$Bee)
par(mfrow=c(5,4),mar=c(1.2,1.4,1,1),oma=c(1.5,1.5,0,0))
#library(ISLR)
for(i in bees) {
window=NULL
```

```
beel= subset(seql,seql$Bee==i)
example=glm(beel$Correct~beel$Visit, family=binomial)
foo=coefficients(summary(example))[8]
plot(beel$Visit, beel$Correct,xlab='Visits',ylim=c(-0.2,1.2),yaxt='n',pch=4,ylab='Correctness',sep='')
ticks<-c(0,0.5,1)
axis(2,at=ticks,labels=ticks,las=1)
text(17,1.15,paste('Bee #',i,', p = ',round(foo, digits = 2)))
pred= predict(example,x=bee15$Visit,type="response")
lines(pred)
for (v in 3:30) { #Adding a 5-visit average
avm=mean(beel$Correct[(v-2):(v+2)])
window=c(window,avm)
abline(h=0.5,col='grey',lty=3)
}
window=cbind(window,c(3:30))
lines(window[,2],window[,1],col='darkgrey',pch=4,lty=2)
}


###---Miscellaneous extra analyses
#Are bees biased to one of the two patterns? No.
par(mfrow=c(1,1))
biases=NULL
bees = unique(seql$Bee)
for (i in bees) {
bee=subset(seql,seql$Bee==i)
beess=subset(bee,bee$First_choice=='R')
countedS=nrow(beess)
biases=c(biases,countedS)}
hist(biases) #it's also normally distributed around 16 and all are within chance levels

# Power analysis of Logistic regressions
#create a series of data poitns which have a logistic curve starting at 50% and ending at 65% and tipping point at 12 visits
seqexb=c(0.5,0.5,0.5,0.502,0.506,0.512,0.52,0.53,0.54,0.55,0.56,0.57,0.58,0.59,0.599,0.608,0.617,0.625,0.631,0.637,0.64,0.64,0.64,0.64,0.64,0.64,0.64,0.64,0.64,0.64,0.64,0.64)
finalprob=0.67#this number can be changed to any desired probability between 0.5 and 1, which determines maximum learning.
seqex=((seqexb-0.5)*((finalprob-0.50)/(0.64-0.50)))+0.50
simulps=NULL
cycles=10 #number of cycles for the simulation to run
for(x in 1:cycles) {
simul=NULL
for(i in 1:20) {
simulp=NULL
for(j in 1:32) {
simulp=c(simulp,rbinom(prob=seqex[j],size=1,n=1))}
simul=as.data.frame(rbind(simul,cbind(rep(i,32),c(1:32),simulp)))}
colnames(simul)=c('Bee','Visit','Correct')
simul$Bee=as.factor(simul$Bee)
thistest=summary(glmer(Correct ~ Visit + (Visit|Bee), family=binomial,data=simul))
simulp=thistest$coefficients[8]
```

```
simulps=c(simulps,simulp)
print(x)}
plot(simulps)
(cycles-sum(simulps>0.05))/cycles #0.70-0.80 power for 0.64 success rate within 22 visits
seqexam=simul
par(mfrow=c(1,2))
listperex1=NULL
for(o in 1:20) {
beet=subset(seqexam,seqexam[,1]==o )
beet=subset(beet, beet[,2] <17)
mus=(sum(beet[,3])/16)
listperex1=c(listperex1,mus)}
listperex2=NULL
for(o in 1:20) {
beet=subset(seqexam,seqexam[,1]==o )
beet=subset(beet, beet[,2] >16)
mus=(sum(beet[,3])/16)
listperex2=c(listperex2,mus)}
par(mfrow=c(1,3))
plot(listperex1,ylim=c(0,1),pch=19,las=1,ylab='Average correct choices',xlab='Bee',main='Overall correct choice for each bee')
abline(h=0.5,col='darkgrey')
t.test(listperex1-0.5)
plot(listperex2,ylim=c(0,1),pch=19,las=1,ylab='Average correct choices',xlab='Bee',main='Overall correct choice for each bee')
abline(h=0.5,col='darkgrey')
t.test(listperex2-0.5)
boxplot(listperex1,listperex2)

##...Figure 2 but individual bees' data are shown
par(mfrow=c(2,3))
plot(listper1,ylim=c(0,1),pch=19,las=1,ylab='Average correct choices',xlab='Bee',main='Overall correct choice for each bee')
abline(h=0.5,col='darkgrey',lty=3)
plot(listpern2,ylim=c(0,1),pch=19,las=1,ylab='Average correct choices',xlab='Bee',main='Overall correct choice for each bee')
abline(h=0.5,col='darkgrey',lty=3)
plot(listperp1,ylim=c(0,1),pch=19,las=1,ylab='Average correct choices',xlab='Bee',main='Overall correct choice for each bee')
abline(h=0.5,col='darkgrey',lty=3)
plot(listper2,ylim=c(0,1),pch=19,las=1,ylab='Average correct choices',xlab='Bee',main='Overall correct choice for each bee')
abline(h=0.5,col='darkgrey',lty=3)
plot(listpern1,ylim=c(0,1),pch=19,las=1,ylab='Average correct choices',xlab='Bee',main='Overall correct choice for each bee')
abline(h=0.5,col='darkgrey',lty=3)
plot(listperp2,ylim=c(0,1),pch=19,las=1,ylab='Average correct choices',xlab='Bee',main='Overall correct choice for each bee')
abline(h=0.5,col='darkgrey',lty=3)

#range of correct choices and binomial tests for each bee
par(mfrow=c(1,1))
```

```
successes=NULL
for(i in corres) {successes=c(successes,as.numeric(binom.test(i,32)[3]))}
plot(successes,pch=19)
range(successes)


#Do bees stick to a pattern?
bees = unique(seql$Bee)
par(mfrow=c(5,4),mar=c(1.2,1.4,1,1),oma=c(1.5,1.5,0,0))
#library(ISLR)
for(i in bees) {
window=NULL
beel= subset(seql,seql$Bee==i)
plot(beel$Visit, as.numeric(beel$First_choice),xlab='Visits',ylab='Rings -- Spokes',yaxt='n',pch=4)
text(2.5,2.5,paste('Bee #',i,sep=''))
text(1.4,2.1,'Ring'); text(1.9,2.9,'Spoke')
for (v in 3:30) { #Adding a 5-visit average
avm=mean(as.numeric(beel$First_choice[(v-2):(v+2)]))
window=c(window,avm)
abline(h=0.5,col='grey',lty=3)
}
window=cbind(window,c(3:30))
lines(window[,2],window[,1],col='darkgrey',pch=4,lty=2)
}

#comparing Salt Vs. Water neutral stimulus in the first and second halves of trials
t.test(listper[1:14],listper[15:20])
t.test(listper1[1:14],listper[15:20])
t.test(listper2[1:14],listper2[15:20])
```

JMP CODES

Code for GLMM analysis of sequence data

```
Fit Model(
	Y( :Correct ),
	Effects( :Visit ),
	Random Effects( :Bee ID ),
	NoBounds( 1 ),
	Personality( "Generalized Linear Mixed Model" ),
	Generalized Distribution( "Binomial" ),
	Link Function( "Logit" ),
	Run( Fit )
);
```

Code for GLMM analysis of sequence data comparing water vs. salt solution treatment

```
Fit Model(
	Y( :Correct ),
	Effects(
		:Visit, :"Non-rewarding treatment"n, :Visit * :"Non-rewarding treatment"n
```

```
    ),
    Random Effects( :Bee ID ),
    Personality( "Generalized Linear Mixed Model" ),
    Generalized Distribution( "Binomial" ),
    Link Function( "Logit" )
);
```

Code for the analysis of the number of times a bee explored the feeders before landing to feed. Exploration data is included in the Zenodo data file.

```
Fit Model(
    Y(
        :Number of times explored before landing to feed,
        :Log number of times explored
    ),
    Effects( :Visit ),
    Random Effects( :Bee ID ),
    Personality( "Standard Least Squares" ),
    Emphasis( "Effect Leverage" ),
    Method( "REML" )
);
```
